# Draft genome sequence of the extremely halophilic archaeon *Haladaptatus cibarius* type strain D43^T^ isolated from fermented seafood

**DOI:** 10.1186/s40793-015-0051-8

**Published:** 2015-08-13

**Authors:** Hae-Won Lee, Dae-Won Kim, Mi-Hwa Lee, Byung-Yong Kim, Yong-Joon Cho, Kyung June Yim, Hye Seon Song, Jin-Kyu Rhee, Myung-Ji Seo, Hak-Jong Choi, Jong-Soon Choi, Dong-Gi Lee, Changmann Yoon, Young-Do Nam, Seong Woon Roh

**Affiliations:** Biological Disaster Analysis Group, Korea Basic Science Institute, Daejeon, 305-806 Republic of Korea; World Institute of Kimchi, Gwangju, 503-360 Republic of Korea; Systems Biology Team, Center for Immunity and Pathology, Korea National Institute of Health, Cheongju, 361-951 Republic of Korea; Research Group of Gut Microbiome, Korea Food Research Institute, Sungnam, 463-746 Republic of Korea; ChunLab Inc., Seoul National University, Seoul, 151-742 Republic of Korea; Department of Food Science and Engineering, Ewha Womans University, Seoul, 120-750 Republic of Korea; Division of Bioengineering, Incheon National University, Incheon, 406-772 Republic of Korea

**Keywords:** Extremely halophilic archaea, *Haladaptatus cibarius*, Genome sequence, Salt-fermented seafood, Glycine betaine, Trehalose

## Abstract

An extremely halophilic archaeon, *Haladaptatus cibarius* D43^T^, was isolated from traditional Korean salt-rich fermented seafood. Strain D43^T^ shows the highest 16S rRNA gene sequence similarity (98.7 %) with *Haladaptatus litoreus* RO1-28^T^, is Gram-negative staining, motile, and extremely halophilic. Despite potential industrial applications of extremely halophilic archaea, their genome characteristics remain obscure. Here, we describe the whole genome sequence and annotated features of strain D43^T^. The 3,926,724 bp genome includes 4,092 protein-coding and 57 RNA genes (including 6 rRNA and 49 tRNA genes) with an average G + C content of 57.76 %.

## Introduction

The extremely halophilic archaea, called haloarchaea, possess the small retinal protein halorhodopsin [1–3] and currently consists of more than 47 genera that live in hypersaline environments [4, 5]. Three members of the genus *Haladaptatus*—*H. paucihalophilus* [6], *H. litoreus* [7], and *H. cibarius* [8]—were isolated from a low-salt, sulfide-rich spring; marine solar saltern; and salt-fermented seafood, respectively. *Haladaptatus* comprises Gram-negative staining, non-motile haloarchaea that have polar lipids including phosphatidylglycerol, phosphatidylglycerol phosphate methyl ester, and phosphatidylglycerol sulfate [6]. The genomic analysis revealed that *H. paucihalophilus* survives in low salinity conditions because of trehalose synthesis with OtsAB pathway and trehalose glycosyl-transferring synthase pathway, and glycine betaine uptake [9]. However, other members in the genus *Haladaptatus* have not been analyzed at the genome level.

*H. cibarius* was isolated from the traditional Korean salt-fermented seafood, which is made with shellfish [8]. D43^T^ (= DSM 19505^T^ = JCM 15962^T^) is a representative strain and designated as the type strain of the species. It can grow in 10%–30% (w/v) NaCl (optimum, 15%), with Mg^2+^ required for growth. In addition, cells are not lysed in distilled water. The genome sequences of this genus are expected to provide fundamental information for the halotolerant features and biotechnological applications of the haloarchaea. Here, we describe the first whole genome sequence of *H*. c*ibarius* along with its annotated features, and summarize the taxonomic classification.

## Organism information

### Classification and features

**Fig. 1:**
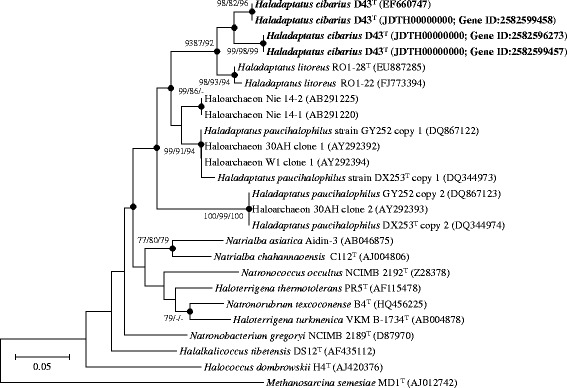
Phylogenetic tree constructed using the neighbor-joining method based on 16S rRNA gene sequences, showing the taxonomic position of strain D43^T^ in genus *Haladaptatus*. Bootstrap values (>70%) at nodes are shown as percentages calculated using the neighbor-joining/maximum parsimony/maximum likelihood probabilities based on 1000 replicates. Filled circles indicate identical branches generated using three algorithms. *Methanosarcina semesiae* MD1^T^ was used as an outgroup. Bar, 0.05 substitutions per nucleotide position

The taxonomic position for *H. cibarius* D43^T^ was identified with type strains obtained from the EzTaxon-e server [10]. The 16S rRNA sequences of D43^T^ and closely related strains were aligned using the ClustalW multiple sequence alignment program [11] and were subsequently used for the phylogenetic analysis. Phylogenetic trees were constructed using the neighbor-joining [12], maximum-parsimony [13], and maximum likelihood [14] algorithms with bootstrap values of 1,000 using MEGA version 5 molecular evolutionary genetics analysis program [15]. Strain D43^T^ clustered with type strains of *Haladaptatus* species (Fig. 1), exhibiting 16S rRNA gene sequence similarities of 98.7% and 95.1% between strain D43^T^ (EF660747) and the type strain of *H. litoreus* and *H. paucihalophilus*, respectively. Classification and general features of *H. cibarius* D43^T^ are shown in Table 1.

**Table 1:** Classification and general features of *Haladaptatus cibarius* D43^T^ [18]

MIGS ID	Property	Term	Evidence code^a^
	Classification	Domain *Archaea*	TAS [24]
		Phylum *Euryarchaeota*	TAS [25]
		Class *Halobacteria*	TAS [26]
		Order *Halobacteriales*	TAS [27, 28]
		Family *Halobacteriaceae*	TAS [28, 29]
		Genus *Haladaptatus*	TAS [6]
		Species *Haladaptatus* c*ibarius*	TAS [8]
		Type strain D43^T^ (DSM 19505, JCM 15962)	TAS [8]
	Gram stain	Negative	TAS [8]
	Cell shape	coccus or coccobacillus	TAS [8]
	Motility	motile	TAS [8]
	Sporulation	Not reported	TAS [8]
	Temperature range	15–50 °C	TAS [8]
	Optimum temperature	37 °C	TAS [8]
	pH range; Optimum	6.0–8.0; 7.0	TAS [8]
	Carbon source	Sucrose, D-fructose, D-glucose, lactose, formate, acetate	TAS [8]
MIGS-6	Habitat	Salt-fermented seafood	TAS [8]
MIGS-6.3	Salinity	35 % NaCl (w/v)	TAS [8]
MIGS-22	Oxygen requirement	Aerobic	TAS [8]
MIGS-15	Biotic relationship	Free-living	TAS [8]
MIGS-14	Pathogenicity	Not reported	
MIGS-23.1	Isolation	Salt-fermented food	TAS [8]
MIGS-4	Geographic location	Republic of Korea	TAS [8]
MIGS-5	Sample collection time	Not reported	
MIGS-4.1	Latitude	Not reported	
MIGS-4.2	Longitude	Not reported	
MIGS-4.3	Depth	Not reported	
MIGS-4.4	Altitude	Not reported	

^**a**^Evidence codes - TAS: traceable author statement (i.e., a direct report exists in the literature). These evidence codes are from the Gene Ontology project [30]

**Fig. 2:**
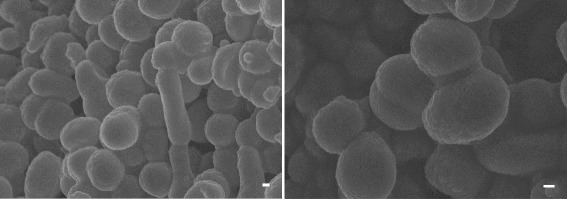
Scanning electron micrographs of *H. cibarius* D43^T^ obtained by SUPRA 55VP (Carl Zeiss, Jena, Germany). Scale bars represent 200 nm

Strain D43^T^ is a Gram-negative staining, coccus or coccobacillus, motile archaeon approximately 1.0 μm in diameter (Fig. 2). Catalase and oxidase tests yielded positive results, but reduction of nitrate to nitrite under aerobic conditions was negative. Cells contained the polar lipids phosphatidylglycerol, phosphatidylglycerol phosphate methyl ester, and two unidentified glycolipids. Strain D43^T^ hydrolyzed gelatin and Tween 80, utilized formate and acetate as carbon sources, and produced acid from sucrose and d-glucose. The strain was sensitive to anisomycin, aphidicolin, chloramphenicol, and rifampicin, and was resistant to ampicillin, erythromycin, kanamycin, streptomycin, and polymycin B.

## Genome sequencing and annotation

### Genome project history

The genome project and sequence of the *H. cibarius* D43^T^ genome were deposited in the Genomes OnLine Database [16] (project ID: Gp0086819) and GenBank (accession number: JDTH00000000), respectively. The BioProject number was PRJNA236630. Sequencing and annotation were performed by Chun Lab Inc. (Seoul, Korea) and Integrated Microbial Genomes Expert Review (IMG-ER) [17].

### Growth conditions and genomic DNA preparation

*H. cibarius* D43^T^ grew optimally on halophilic medium [6] supplemented with 15% (w/v) NaCl and 20 mM Mg^2+^ adjusted to pH 7.0, producing colonies with a pink color after incubation at 37°C as previously described [8]. Genomic DNA was extracted and purified using a G-spin DNA extraction kit (iNtRON Biotechnology Inc., Sungnam, Korea), according to the manufacturer’s instructions.

### Genome sequencing and assembly

Genomic sequences of *H. cibarius* D43^T^ were generated from a total of 9,237,360 quality-filtered reads (710.3-fold coverage) by combining 5,074,634 reads (374.9-fold coverage) obtained from Mi-Seq 300 bp paired-end library (Illumina, San Diego, CA, USA), 4,112,798 reads (292.1-fold coverage) obtained from an Ion Torrent Personal Genome Machine 318v2 chip (Life Technologies, Carlsbad, CA, USA), and 49,928 reads (43.3-fold coverage) obtained from PacBio RS 10 kb library (Pacific Biosciences, Menlo Park, USA). Illumina and PGM data were assembled *de novo* with CLC Genomics Workbench 6.5.1 (CLC bio, Boston, MA, USA) and PacBio data were assembled using the HGAP2 algorithm in SMRT Analysis 2.1 (Pacific Biosciences). Resultant contigs were assembled with CodonCode Aligner 3.7 (CodonCode Corporation, Centerville, MA, USA). Sequences were assembled to 13 scaffolds with an N50 contig size of 985,075 bp; the genome sequencing project information and its associated MIGS version 2.0 compliance levels [18] are shown in Table 2.

**Table 2:** Project information

MIGS ID	Property	Term
MIGS-31	Finishing quality	Improved high-quality draft
MIGS-28	Libraries used	Illumina PE, Ion PGM, and PacBio libraries
MIGS-29	Sequencing platforms	Illumina Mi-seq, Ion PGM, and PacBio RS systems
MIGS-31.2	Fold coverage	374.92 × Illumina; 292.08 × Ion PGM; 43.25 × PacBio
MIGS-30	Assemblers	CLC Genomics Workbench 6.5.1, SMRT Analysis 2.1
MIGS-32	Gene calling method	IMG-ER
	Locus Tag	HL45
	GenBank ID	JDTH0000000
	GenBank Date of Release	June 20, 2014
	GOLD ID	Gi0069860
	BIOPROJECT	PRJNA236630
MIGS-13	Source material identifier	D43^T^
	Project relevance	Environmental and biotechnological

### Genome annotation

The open reading frames of the assembled genome were predicted and annotated using IMG-ER [17], NCBI COG [19], Pfam [20], and EzTaxon-e [10] databases. The rRNA and tRNA genes were identified using RNAmmer 1.2 [21] and tRNA scan-SE 1.23 [22], respectively.

## Genome properties

**Fig. 3:**
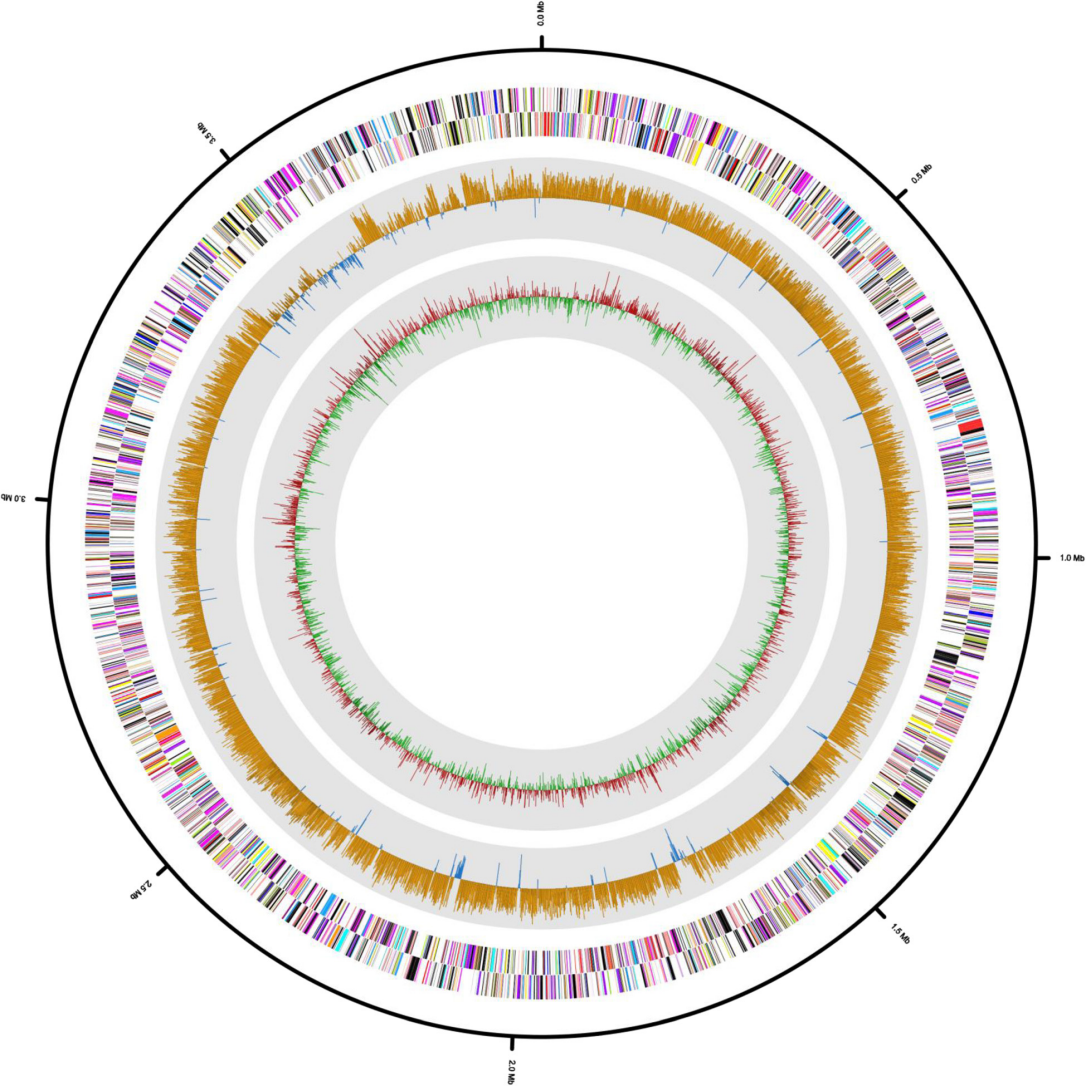
Graphical map of the *H. cibarius* D43^T^ pseudochromosome. From outside to center: RNA genes (red, tRNA and blue, rRNA) and genes on the antisense and sense strands (colored according to COG categories). Inner circle shows the GC skew, with yellow and blue indicating positive and negative values, respectively. GC content is indicated in red and green. The genome map was visualized using CLgenomics 1.06 (Chun Lab Inc.)

The draft genome sequence for *H. cibarius* D43^T^ contained 3,926,724 bp, with 13 scaffolds. The G + C content was 57.76 % (Fig. 3 and Table 3), and 4,092 protein-coding genes were predicted along with 57 RNA genes, including six rRNA (two 5S, three 16S, and one 23S rRNA), 49 tRNA, and two additional RNA genes. There were 2,676 protein-coding genes with predicted functions: 773 were enzymes, 98 encoded signal peptides, and 1,049 encoded transmembrane proteins. The distribution of genes in the COG functional categories is shown in Table 4. A large number of genes were associated with the COG functional categories of cell wall biogenesis (79, 3.3 %); transcription (100, 4.1 %); and transport and metabolism of amino acids (299, 12.3 %), carbohydrates (121, 5.0 %), and lipids (80, 3.3 %). Further analysis with dbCAN [23], a database for annotation of carbohydrate-active enzymes, showed that the genome contains genes encoding various enzymes for the breakdown and biosynthesis of carbohydrates such as chitinase (GH18), chitosanase (GH5), pullulanase (GH13), trehalose synthase (GT4 and 20), cellulose synthase (GT2), and alginate lyase (PL6).

**Table 3:** Genome statistics

Attribute	Value	% of Total
Genome size (bp)	3,926,724	100.00
DNA coding (bp)	3,378,684	86.04
DNA G + C (bp)	2,267,915	57.76
DNA scaffolds	13	100.00
Total genes	4,149	100.00
Protein-coding genes	4,092	98.63
RNA genes	57	1.37
Genes in internal clusters	3,135	75.56
Genes with function prediction	2,676	64.50
Genes assigned to COGs	2,188	52.74
Genes assigned Pfam domains	2,699	65.05
Genes with signal peptides	98	2.36
Genes with transmembrane helices	1049	25.28
CRISPR repeats	4

**Table 4:** Number of genes associated with general COG functional categories

Code	Value	% age	Description
J	164	6.76	Translation, ribosomal structure, and biogenesis
A	1	0.04	RNA processing and modification
K	100	4.12	Transcription
L	102	4.20	Replication, recombination, and repair
B	3	0.12	Chromatin structure and dynamics
D	20	0.82	Cell cycle control, cell division, chromosome partitioning
Y	0	0.00	Nuclear structure
V	37	1.53	Defense mechanisms
T	55	2.27	Signal transduction mechanisms
M	79	3.26	Cell wall/membrane biogenesis
N	28	1.15	Cell motility
Z	0	0.00	Cytoskeleton
W	0	0.00	Extracellular structures
U	28	1.15	Intracellular trafficking and secretion, and vesicular transport
O	88	3.63	Post-translational modification, protein turnover, chaperones
C	162	6.68	Energy production and conversion
G	121	4.99	Carbohydrate transport and metabolism
E	299	12.32	Amino acid transport and metabolism
F	76	3.13	Nucleotide transport and metabolism
H	109	4.49	Coenzyme transport and metabolism
I	80	3.30	Lipid transport and metabolism
P	173	7.13	Inorganic ion transport and metabolism
Q	46	1.90	Secondary metabolism biosynthesis, transport, and catabolism
R	392	16.16	General function prediction only
S	263	10.84	Function unknown
-	1961	47.26	Not in COGs

The total is based on the total number of protein coding genes in the genome

## Insights from the genome sequence

The genome analysis of *H. cibarius* D43^T^ revealed genes involved in glycine betaine synthesis—including betaine aldehyde dehydrogenase, glycine betaine demethylase, and choline-glycine betaine transporter gene—that allow *H. cibarius* to maintain osmotic balance in hypersaline environments. In addtion, trehalose-related genes of trehalose-6-phosphate synthase, trehalose-6-phosphatase, trehalose-6-phosphate synthase and trehalose-6-phosphate hydrolase, and trehalose-utilization protein genes were analyzed in the genome sequences of *H. cibarius* D43^T^. The genes related with trehalose synthesis in the genome show the possibility of trehalose production that is important in food industry.

## Conclusions

The draft genome sequences of the extremely halophilic archaeon isolated from the salt-fermented seafood were analyzed. Genes related with glycine betaine and trehalose for the survival in extreme environments were identified. The extremely halophilic archaeon could be a valuable resource for biotechnological applications because hypersaline conditions minimize the risk of contamination by other microorganisms. Further characterization of halophilic enzymes of the haloarchaea based on the genomic analyses can provide more detailed information on enzyme structures and potential industrial applications.

## Abbreviations

PGM: Personal genome machine
IMG-ER: Integrated microbial genomes expert review
ORF: Open reading frame
